# Carbon monoxide down-regulates α4β1 integrin-specific ligand binding and cell adhesion: a possible mechanism for cell mobilization

**DOI:** 10.1186/s12865-014-0052-1

**Published:** 2014-10-31

**Authors:** Alexandre Chigaev, Yelena Smagley, Larry A Sklar

**Affiliations:** Department of Pathology and University of New Mexico Cancer Center, Albuquerque, NM 87131 USA; University of New Mexico Center for Molecular Discovery, Albuquerque, NM 87131 USA; University of New Mexico Health Sciences Center, Albuquerque, NM 87131 USA

**Keywords:** Carbon monoxide, Hemin, Integrin, Affinity, Conformation, Cell adhesion

## Abstract

**Background:**

Carbon monoxide (CO), a byproduct of heme degradation, is attracting growing attention from the scientific community. At physiological concentrations, CO plays a role as a signal messenger that regulates a number of physiological processes. CO releasing molecules are under evaluation in preclinical models for the management of inflammation, sepsis, ischemia/reperfusion injury, and organ transplantation. Because of our discovery that nitric oxide signaling actively down-regulates integrin affinity and cell adhesion, and the similarity between nitric oxide and CO-dependent signaling, we studied the effects of CO on integrin signaling and cell adhesion.

**Results:**

We used a cell permeable CO releasing molecule (CORM-2) to elevate intracellular CO, and a fluorescent Very Late Antigen-4 (VLA-4, α_4_β_1_-integrin)-specific ligand to evaluate the integrin state in real-time on live cells. We show that the binding of the ligand can be rapidly down-modulated in resting cells and after inside-out activation through several Gα_i_-coupled receptors. Moreover, cell treatment with hemin, a natural source of CO, resulted in comparable VLA-4 ligand dissociation. Inhibition of VLA-4 ligand binding by CO had a dramatic effect on cell-cell interaction in a VLA-4/VCAM-1-dependent cell adhesion system.

**Conclusions:**

We conclude that the CO signaling pathway can rapidly down-modulate binding of the VLA-4 -specific ligand. We propose that CO-regulated integrin deactivation provides a basis for modulation of immune cell adhesion as well as rapid cell mobilization, for example as shown for splenic monocytes in response to surgically induced ischemia of the myocardium.

## Background

Since the discovery that endogenous CO can serve as a neurotransmitter [1] and that it exhibits anti-inflammatory properties [2] the number of papers devoted to CO signaling and therapeutic applications has increased every year [3-6]. Since the roles of the two major gaseous messengers, CO and nitric oxide (NO) are somewhat similar [7], and our recent discovery of rapid effects of NO on integrin ligand binding affinity and cell adhesion [8], we studied the effects of CO on integrin regulation.

Integrins are cell adhesion receptors that are capable of modulating rapid adhesion and de-adhesion events, without a change in the number of molecules expressed [9]. Ligand interactions with integrins represent the molecular basis of integrin-dependent cell adhesion. Integrin-dependent cell adhesion is controlled by the conformational state of the molecule through ligand binding affinity and extension that are regulated by a number of signaling pathways initiated by other cellular receptors. This so-called inside-out signaling serves as the basis for rapid leukocyte arrest on endothelium, cell migration and chemotaxis, mobilization, trafficking, and interaction of immune cells [10,11]. α_4_β_1_-integrin (CD49d/CD29, Very Late Antigen-4, VLA-4) is expressed on leukocytes, dendritic cells, hematopoietic progenitors, and stem cells, as well as cancer cells of differing origin [12,13]. The goal of the current study was to examine the effects of the CO signaling pathway on VLA-4 conformational regulation.

To date, a limited number of studies have been dedicated to the effects of CO on leukocyte integrin-dependent adhesion. The down-regulation of leukocyte extravasation and leukocyte endothelial cell interaction, as well as the effects on integrin or integrin ligand expression have been reported [14-19]. To the best of our knowledge, this report represents the first time that the effects of CO and a natural source of CO, hemin, on integrin ligand binding have been studied on live cells in real-time under inside-out signaling conditions. Our findings provide a molecular mechanism for inhibition of integrin-dependent leukocyte adhesion and the beneficial effects of CO-associated therapies in a number of pathologies [4].

## Results

### Carbon monoxide signaling and pathway modulation

**Figure 1:**
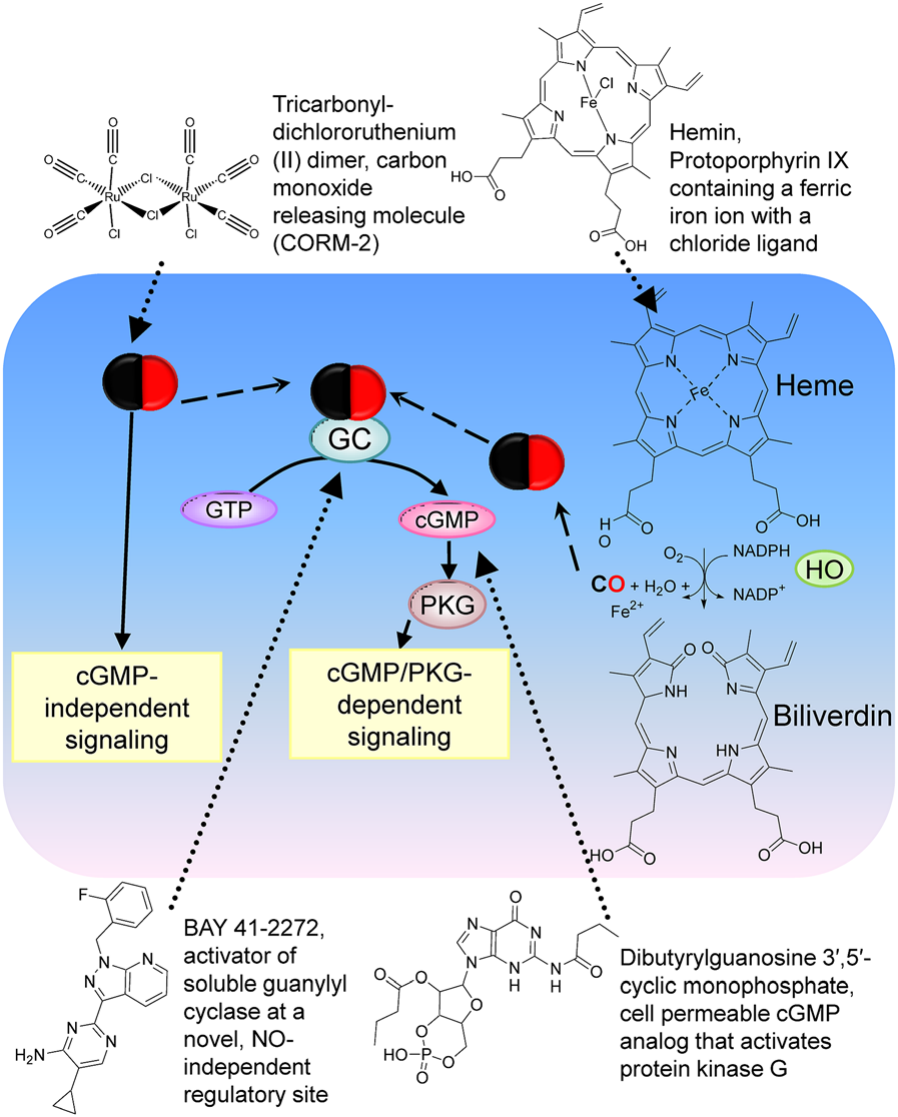
**CO signaling cascade and small molecules used to modulate this pathway.** Endogenous carbon monoxide is generated by heme oxygenase (HO). In cells CO reacts with hemeproteins. One of the CO receptors is the guanylyl cyclase (GC) that produces the intracellular messenger cyclic GMP (cGMP). cGMP interacts with the cGMP-dependent protein kinase (PKG), which phosphorylates multiple substrates, and participates in signal propagation. The carbon monoxide donor provides an exogenous source of CO. The natural HO substrate hemin increases CO production because conversion of heme into biliverdin represents the rate-limiting step in heme degradation [5]. The activator of soluble guanylyl cyclase binds to GC, and induces enzyme activation in the absence of CO. The cell permeable analog of cGMP diffuses across the plasma membrane, and thus, activates cGMP-dependent signaling.

Endogenous CO is produced inside cells as a product of enzymatic degradation of heme catalyzed by the enzyme heme oxygenase (HO) that consists of two isoforms: HO-1 is inducible, and HO-2 is a constitutive form [20]. HO is localized in the cytosol and mitochondria [21]. The major source of heme for CO production is hemoglobin, and other hemeproteins (Figure 1). CO has been shown to stimulate soluble guanylyl cyclase, stimulate vasodilatation, block cell proliferation, participate in neural transmission, and inhibit platelet aggregation (see [20] and references therein). CO targets several heme-containing proteins and soluble guanylyl cyclase (sGC) was recognized as the first and the most studied “CO receptor”. Multiple physiological effects including vasodilatation and platelet aggregation are attributed to the cGMP/PKG-dependent arm of the CO signaling pathway. Other signaling molecules such as p38 MAPK, ERK1/2, and JNK can be regulated through other “CO sensors” [20,22].

To study the effects of CO/cGMP signaling in leukocytes, we selected several small molecules that specifically target this pathway (Figure 1). Tricarbonyldichlororuthenium (II) dimer, Carbon Monoxide-Releasing Molecule 2 (CORM-2), can be described as a complex of a transition metal ruthenium with carbon monoxide [23]. It spontaneously releases CO and is used as a carbon monoxide donor. It exerts a vasodilatory effect in isolated blood vessels, [24]. CORM-2 *in vivo* is shown to diminish adhesion and accumulation of PMNs in injured mice [25,26]. Hemin is a heme substrate analog and an inducer of HO-1 expression [19]. The conversion of one molecule of heme into biliverdin results in the release of one molecule of CO. The HO enzyme that generates CO from hemin is expressed in our model system, and hemin addition has been shown to decrease pro-inflammatory cytokine levels in U937 cells [27]. BAY 41–2272 is an activator of soluble guanylyl cyclase, which was shown to stimulate cGMP production [28]. N^2^,2’-O-dibutyrylguanosine 3’,5’-cyclic monophosphate is a cell permeable cGMP analog that activates protein kinase G [29]. All these molecules are shown to stimulate different steps of the signaling pathway (Figure 1), and therefore, are used to mimic CO-dependent signaling.

### The CO donor induces a rapid decrease in the binding of the VLA-4 specific ligand

**Figure 2:**
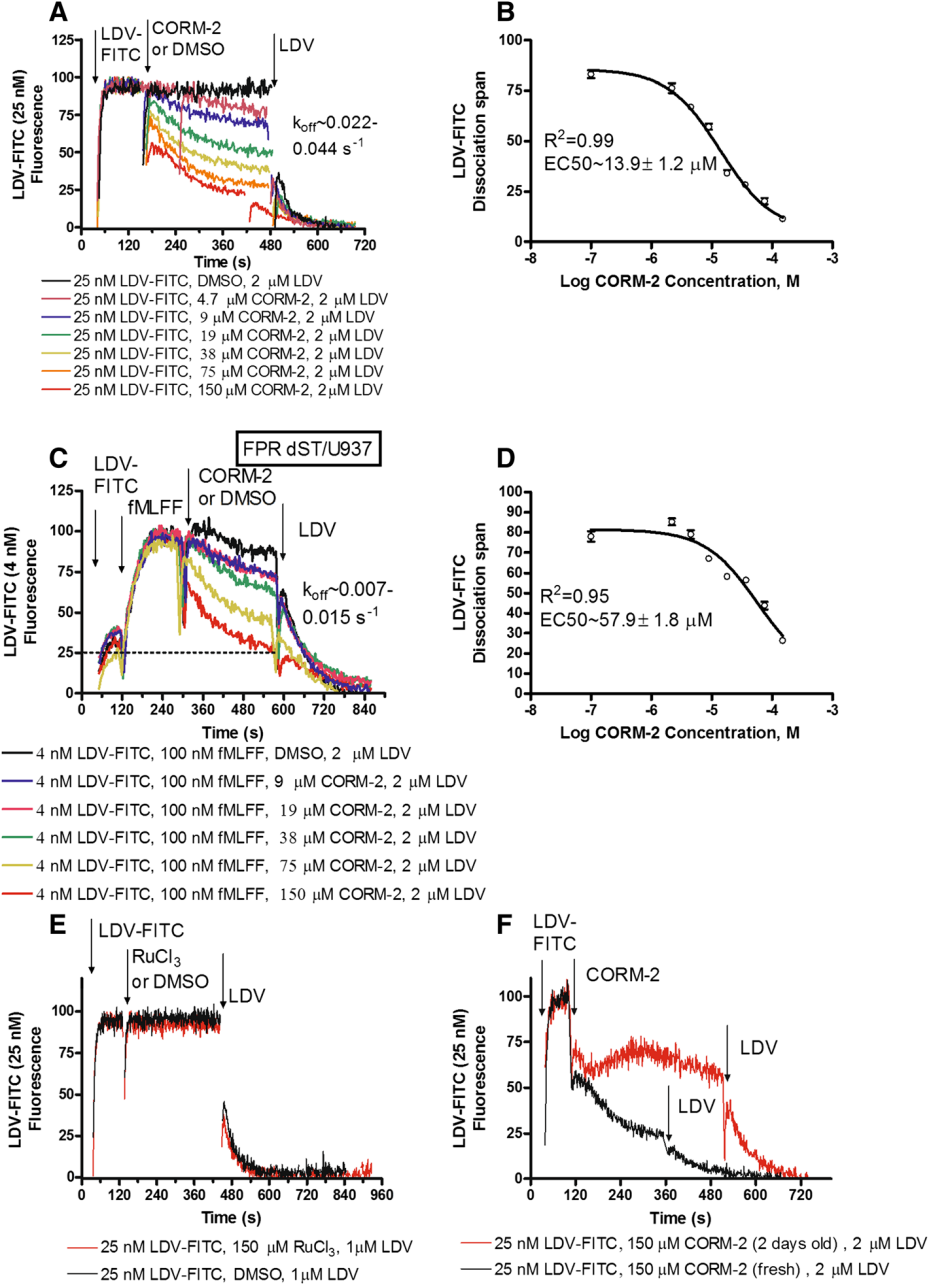
**Effect of CO donor on binding and dissociation of the LDV-FITC probe on resting and activated cells.** LDV-FITC binding and dissociation on U937 cells stably transfected with the non-desensitizing mutant FPR ΔST plotted as LDV-FITC fluorescence *versus* time. The data were normalized to the level of the non-specific signal determined by addition of excess unlabeled competitor (LDV), and therefore, no autofluorescence can be seen. **A**. The experiment involved sequential additions of the LDV-FITC, and different concentrations of CORM-2 or vehicle. The non-specific binding of the probe was determined using LDV. Ligand dissociation rates (k_off_) were determined by fitting the dissociation part of the curves to the single exponential equation. **B**. The span of the single exponential fits for the dissociation curves (from A after LDV addition) plotted *versus* logarithm of CORM-2 concentration. Means ± SEM of two independent determinations are shown. The sigmoidal dose–response (Hill slope = 1) was fit using GraphPad Prism. **C**. The sequential addition of the LDV-FITC, the high affinity FPR ligand (fMLFF), CORM-2 or vehicle, and LDV. LDV-FITC k_off_s were determined as described for A. The level of LDV-FITC binding corresponding to resting cells is indicated by the dashed line. **D**. The span of single exponential fits for the curves (from panel **C**) plotted *versus* logarithm of CORM-2 concentration. Means ± SEM of two independent determinations are shown. The dose–response was fit analogously to B. **E**. The experiment involved addition of the LDV-FITC, RuCl_3_ or vehicle. **F**. The sequential addition of the LDV-FITC, and CORM-2. The “old” CORM-2 was prepared by incubating the solution for 48 hours at room temperature. The non-specific binding of the LDV-FITC probe was determined using LDV. For panels **A**, **C**, **E**, and **F**, a representative experiment of two independent experiments is shown.

The VLA-4-specific ligand (LDV-FITC) is a small fluorescent probe based on the published structure of BIO1211, a CD49d/CD29 specific antagonist [30-32]. The molecule contains the Leu-Asp-Val (LDV) ligand binding motif from the alternatively spliced connecting segment-1 (CS-1) peptide of fibronectin. The major advantage of this probe is that it can be used to detect VLA-4 conformational changes on live cells in real-time in response to cell signaling [8,33,34]. The binding affinity detected using LDV-FITC varies in parallel with VCAM-1, the major natural VLA-4 ligand [35]. VCAM-1 contains the Ile-Asp-Ser (IDS) motif homologous to LDV, and VLA-4 interaction with VCAM-1 can be blocked by LDV-containing molecules [35-37]. To determine the effect of the CO donor on resting cells, samples were first treated with 25 nM LDV-FITC (Figure 2A). This concentration is about 2 fold higher than the dissociation constant for LDV-FITC binding to U937 cells without activation (K_d_ ~12 nM, [30]). Therefore, 70–80% of low affinity sites are occupied. Next, the addition of CORM-2 resulted in the dose-dependent dissociation of LDV-FITC that reached a steady-state 5–6 min after addition. Finally, an excess of unlabeled competitor (LDV) was added to determine the non-specific binding of the probe (Figure 2A). This induced rapid LDV-FITC dissociation with a rate (k_off_) similar to the rate reported for resting cells [35]. To determine the EC50 for the effect of CORM-2 on LDV-FITC binding, the span of the single exponential fits for the dissociation curves after LDV addition was plotted *versus* the logarithm of CORM-2 concentration (Figure 2B).

Next, to study the effect of the CO donor on cells activated through the “inside-out” signaling pathway, we used U937 cells stably transfected with the non-desensitizing mutant of the formyl peptide receptor (FPR). Because serine and threonine in the C-terminal tail of GPCRs phosphorylated by G protein-coupled receptor kinases upon ligation are critical for the binding of arrestin and receptor desensitization [38,39], we used a mutant FPR lacking all serines and threonines (FPR ΔST) [40]. After ligation of this receptor with a high affinity ligand N-formyl-Met-Leu-Phe-Phe FPR signaling persists and the high affinity of the VLA-4 ligand binding pocket is maintained for thousands of seconds [8,34].

To observe real-time inside-out VLA-4 activation, cells were first treated with 4 nM LDV-FITC (Figure 2C). This concentration is below the dissociation constant (K_d_) for its binding to resting VLA-4 (low affinity state, K_d_ ~12 nM), and above the K_d_ for physiologically activated VLA-4 (high affinity state, K_d_ ~1-2 nM) [30]. Thus, the transition from the low affinity to the high affinity receptor state led to increased binding of the probe (from ~25% to ~70-80% of receptor occupancy) [41]. Therefore, the binding of additional LDV-FITC molecules is detected as a rapid increase in the cell fluorescence. Because the laser of the flow cytometer excites only a small volume of solution around the cell, the detection of probe binding is possible in a homogeneous (no-wash) format [42,43]. Next, the addition of CORM-2 resulted in the dose-dependent dissociation of LDV-FITC. Excess unlabeled competitor (LDV) was used to determine the non-specific binding of the probe (Figure 2C). The LDV-FITC dissociation rate (k_off_) was slower but similar to the rate reported for cells activated through inside-out GPCR signaling [30,35]. The EC50 for the effect of CORM-2 on activated cells was somewhat higher than for resting cells (Figure 2D).

To establish whether the effect of CORM-2 was mediated by CO, we conducted two control experiments (Figure 2E, F). First, cells were treated with RuCl_3_ at a concentration equal to the highest concentration used of CORM-2 (150 μM). RuCl_3_ is a product of CORM-2 degradation, and therefore, it was used as a negative control in CORM-2 experiments [44-47]. No statistically significant effects of RuCl_3_ on LDV-FITC binding or dissociation were detected (Figure 2E).

As a second control we took advantage of the limited stability of CORM molecules [48]. First, a stock solution of CORM-2 was prepared and incubated at room temperature for 48 hours. The effect of this old solution was compared to a fresh equimolar CORM-2 solution prepared prior to the experiment (Figure 2F). The freshly prepared solution induced significantly higher dissociation. Thus, because gaseous CO is released into the air over time, the difference between fresh and old CORM-2 solution may be attributed to the lower concentration of CO in the old solution. Based on the two controls we concluded that the effect of CORM-2 is attributed to CO released by the CO donor.

### Effect of the CO donor on the binding of VLA-4 specific ligand during inside-out activation through CXCR4 and CXCR2

**Figure 3:**
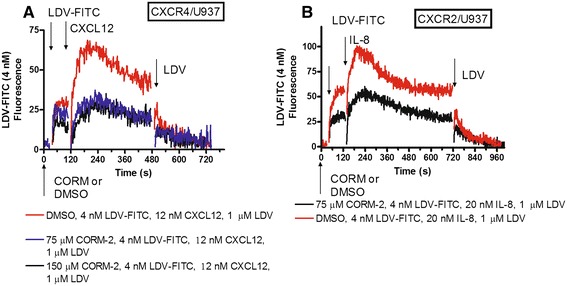
**Effect of CO donor pretreatment on binding and dissociation of the LDV-FITC probe in cells stably transfected with CXCR4 and CXCR2, then treated with different G**α**i-coupled receptor ligands. A**. The experiment involved sequential addition of CORM-2 (75–150 μM, CO donor) or DMSO (control), the fluorescent LDV-FITC probe (4 nM), CXCL12/SDF-1 (12 nM), and excess unlabeled LDV competitor (1 μM). Rapid and reversible binding of the probe reflects the VLA-4 affinity change. **B**. The experiment involved sequential addition of CORM-2 (75μM, CO donor) or DMSO (control), the fluorescent LDV-FITC probe (4 nM), CXCL8/IL-8 (20 nM), and excess unlabeled LDV competitor (1 μM). A representative experiment of two independent experiments is shown.

Cell activation through wild type GPCRs induces a rapid and reversible VLA-4 affinity change due to receptor desensitization [30]. Therefore, in CXCR4 and CXCR2 experiments, the CO donor was added two minutes prior to the addition of GPCR ligands (Figure 3). The treatment of cells stably transfected with wild type CXCR4 or CXCR2 significantly decreased the amplitude of the ligand-induced LDV-FITC response. Thus, similar to a non-desensitizing mutant of FPR the effect of the CO donor can be observed for signaling through other G-protein coupled receptors. This result is similar to the previously reported effect of NO on GPCR-induced VLA-4 activation [8].

### Hemin, a natural substrate of HO and a natural source of CO, rapidly decreases binding of the VLA-4 specific ligand

**Figure 4:**
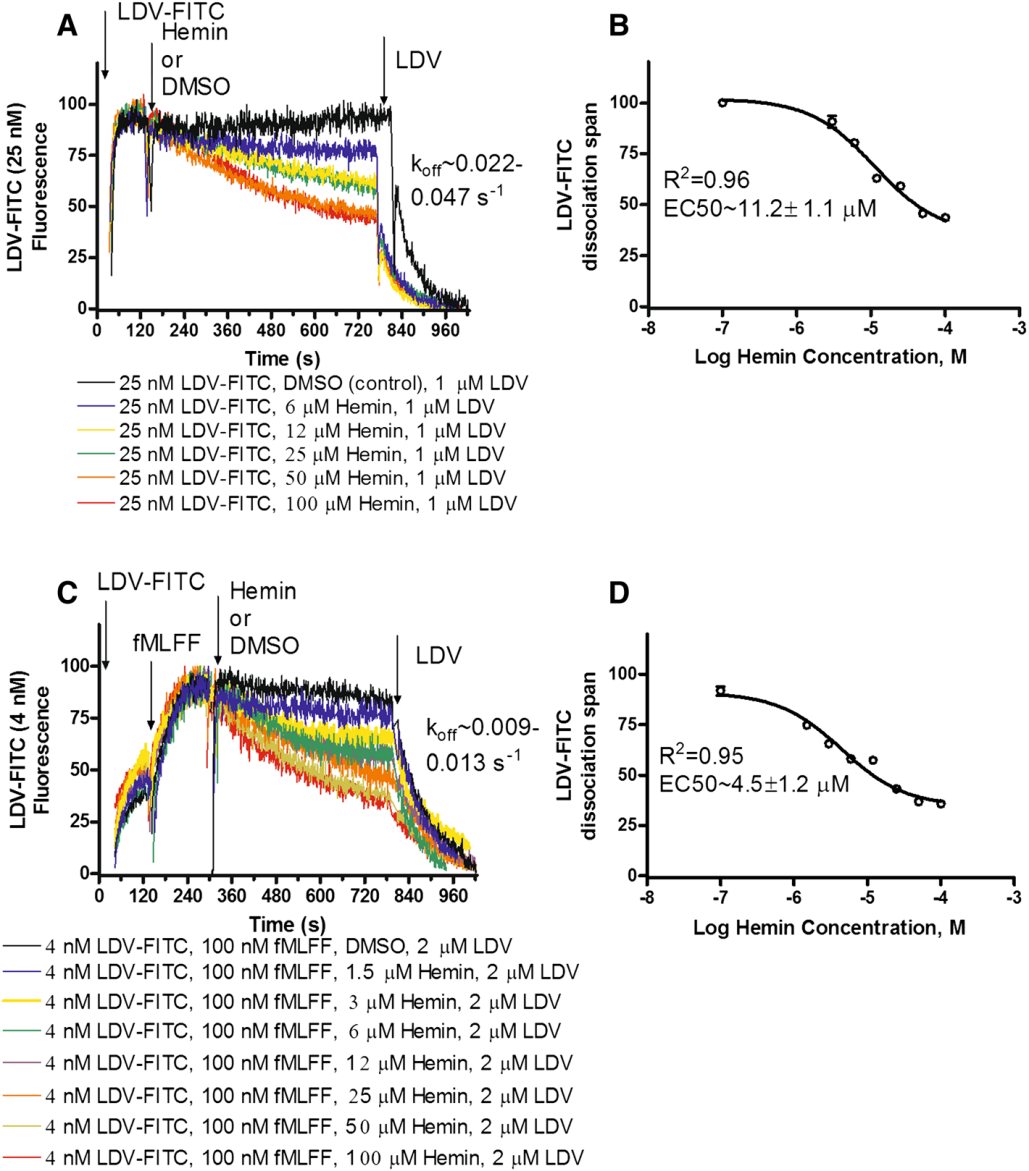
**Effect of hemin on binding and dissociation of the LDV-FITC probe on resting and activated U937 cells.** LDV-FITC probe binding and dissociation on U937 cells plotted as LDV-FITC fluorescence *versus* time. The data were normalized to the level of the non-specific signal determined by the addition of excess unlabeled competitor (LDV 2 μM), and therefore, no cell autofluorescence can be seen. **A**. The experiment involved sequential addition of the fluorescent LDV-FITC probe (25 nM), and different concentrations of hemin (6–100 μM) or DMSO (vehicle). The non-specific binding of the LDV-FITC probe was determined using excess unlabeled competitor (LDV). Ligand dissociation rates (k_off_) were determined by fitting the dissociation part of the curves (after LDV addition) to the single exponential equation. The range of k_off_ is shown. **B**. The span of the single exponential fits for the dissociation curves (from panel A after LDV addition) plotted *versus* logarithm of hemin concentration. Means ± SEM of two independent determinations are shown (n = 2). The sigmoidal dose–response fit (Hill slope = 1) was obtained using GraphPad Prism software. **C**. The experiment was conducted using U937 cells stably transfected with the FPR ΔST receptor, and involved sequential addition of the fluorescent LDV-FITC probe (4 nM), the high affinity FPR ligand N-formyl-Met-Leu-Phe-Phe (100 nM), hemin (1.5-100 μM) or DMSO (control), and LDV (2 μM). LDV-FITC dissociation rates (k_off_) were determined as described for panel A. **D**. The span of the single exponential fits for the dissociation curves (from panel C, after LDV addition) plotted *versus* logarithm of hemin concentration. Means ± SEM of two independent determinations are shown (n = 2). The sigmoidal dose–response fit was obtained analogously to panel **B**.

Heme oxygenase is expressed in U937 cells [49-51], and exogenous hemin can be used to modulate cell signaling in this cell line [27,52]. To study the effect of hemin on LDV-FITC binding on resting and activated cells, experiments were conducted in a manner similar to the CO donor experiments (Figure 2). Resting U937 cells were treated with 25 nM LDV-FITC (Figure 4A). Next, appropriate concentrations of hemin were added. We observed a slow dose-dependent decrease in the LDV-FITC signal. Finally, to determine the non-specific binding of the LDV-FITC probe, excess unlabeled competitor was added 10–12 min later. As in the case of the CO donor, the ligand dissociation rate (k_off_) was similar to the rate reported for resting cells. To determine the EC50 for the effect of hemin on LDV-FITC binding, the span of single exponential fits for the dissociation curves after LDV addition was plotted *versus* the logarithm of hemin concentration (Figure 4B). The effect of hemin was similar to the effect of the CO donor on resting cells.

The effect of hemin on “inside-out” activated cells (Figure 4C) has been studied in a manner analogous to the CO donor experiments (Figure 2C). Cells were treated with LDV-FITC, FPR ligand for cell activation, appropriate concentrations of hemin, and the unlabeled LDV competitor (Figure 4C). Analogous to the CO donor results, the ligand dissociation rate (k_off_) was similar to the rate reported for activated cells, and the EC50 for the effect of hemin on activated cells was comparable to the EC50 for resting cells (Figure 4D). Thus, the natural source of CO, hemin, exhibited activity that was analogous to that of the artificial CO donor. Both compounds induced rapid dissociation of the VLA-4 specific ligand.

### Carbon monoxide donor produces a small effect on VLA-4 subunit surface expression

**Figure 5:**
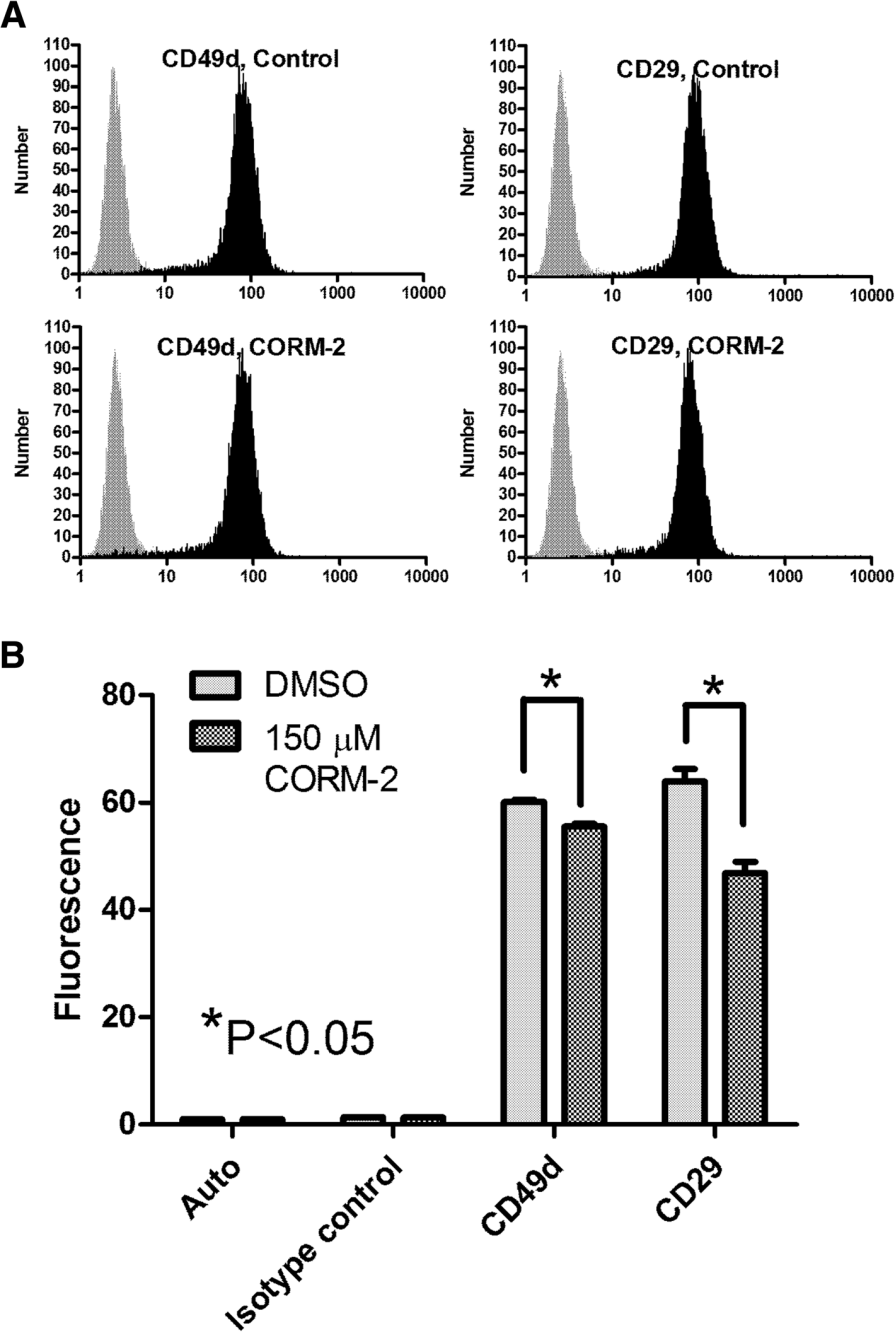
**Effect of CO donor on surface expression of VLA-4 (CD49d/CD29 heterodimer).** U937 cells were treated with vehicle (DMSO, control), and CO donor (150 μM CORM-2) for 30 min at 37°C. Next, cells were placed on ice and stained with primary labeled anti-CD29 and anti-CD49d antibodies, or the isotype control. **A**. Histograms of anti-CD29 and anti-CD49d antibodies are shown in black, and the isotype control is grey. **B**. Bar graphs of mean channel fluorescence (MCF) ± SEM (n =5) for unstained cells (autofluorescence), nonspecific binding to cells (isotype control), cells treated with vehicle (DMSO), and cells treated with CORM-2 are shown (10,000 gated events for each sample were collected). One representative experiment of three experiments is shown. * indicates means are significantly different (P <0.05) as estimated by the unpaired t test analysis using GraphPad Prism software.

To study the effect of CO signaling on VLA-4 surface expression, cells were treated with the CO donor or vehicle for 30 min at 37°C. Next, cells were stained with primary labeled antibody against α4- and β1-integrin subunits (Figure 5). Analysis of antibody binding revealed a decrease in the surface expression of both integrin subunits that varied from 10% to 26% in multiple experiments. Given the small sample to sample variation this difference was statistically significant (Figure 5B). However, this difference in the expression of VLA-4 subunits detected after 30 min of CO donor treatment cannot account for rapid and dramatic decrease in the VLA-4-specific ligand binding that has been detected after CO donor addition (Figure 2B,C).

### Carbon monoxide donor diminishes VLA-4 /VCAM-1-dependent cell adhesion

**Figure 6:**
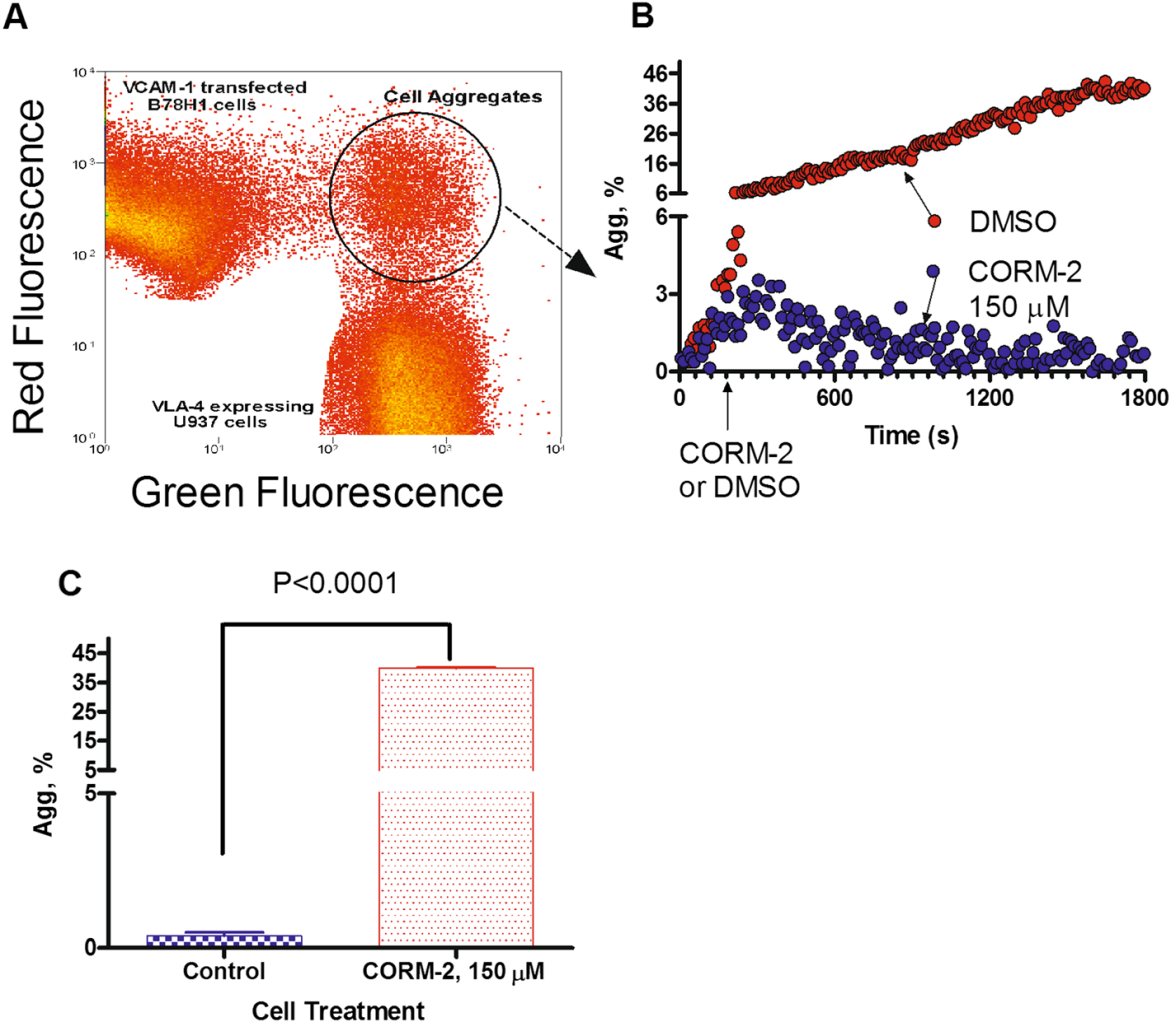
**Effect of CO donor on cell adhesion between U937 cells and VCAM-1-transfected B78H1 cells. A**. Dot plot of flow cytometric analysis of cell aggregation. Cells were labeled with red and green fluorescent dyes. Next, cells were mixed at 0 time point. During data acquisition samples were maintained at 37°C, and continuously stirred with a magnetic stir bar. An increase in the number of aggregates was detected as green and red co-fluorescent particles indicated by the circular gate. **B**. Real-time cell aggregation plotted as % aggregates (Agg, %) *versus* time. The data were normalized to the non-specific aggregation determined as cell aggregation in the presence of excess unlabeled competitor (1 μM LDV). A representative experiment out of two experiments is shown. **C**. Statistical significance of the CO donor effect on cell aggregation. The aggregate percentage data from the last 5 min of the experiments (B) are compared using the unpaired t test. Means are significantly different (P < 0.05).

Next, to study the implications of the CO signaling pathway on integrin-dependent cell adhesion, we utilized a VLA-4/VCAM-1-specific real-time cell aggregation assay [35]. The specificity of cell aggregation in this model system was tested using anti-α4-integrin blocking mAb as well as unlabeled LDV that completely blocked cell aggregation [35,53]. Prior to mixing, U937 cells constitutively expressing VLA-4 were labeled with green fluorescent dye, and B78H1 cells stably transfected with human VCAM-1 were stained with red fluorescent dye (Figure 6A). The cell aggregates were detected as red and green co-fluorescent events in real-time. Cells were mixed and the baseline aggregation data were collected for the first three minutes. Next, the tube was removed and an aliquot of stock solution of carbon monoxide donor CORM-2 in DMSO or equal volume of vehicle (DMSO) were added. The tube was rapidly replaced and data acquisition was reestablished. The data were collected for up to 30 min (Figure 6B).

The CO donor treatment was sufficient to fully reverse cell aggregation (Figure 6B). During the first three minutes of the experiment no significant difference between the two samples was detected. After the addition of CORM-2, cells continued to aggregate for ~2 minutes, and then aggregate accumulation stopped and disaggregation occurred. The sample treated with vehicle exhibited a long-term accumulation of cell aggregates that reached 30-40% by the end of the experiment (Figure 6B). The difference between samples treated with the CO donor and untreated samples was statistically significant (Figure 6C). Thus, the CO donor added to the cell suspension in real-time prevented VLA-4/VCAM-1-dependent aggregation.

### The carbon monoxide donor decreases the binding affinity of the VLA-4 specific ligand

Next, to quantify the effect of CO on VLA-4 specific ligand binding affinity, we evaluated the binding of unlabeled LDV ligand using a Ligand Induced Binding Site (LIBS) antibody. Binding of ligands to integrins induces a series of conformational changes that result in the exposure of previously hidden epitopes. A LIBS antibody can recognize these epitopes. This mAb feature can be used to detect ligand occupied integrin receptors. The parental ligand molecule (BIO1211) [32], and the LDV ligand are known to induce LIBS epitopes [41,54]. To determine the ligand binding affinity (K_d_) for the unlabeled molecule, cells are incubated with increasing concentrations of the ligand in the presence of a constant concentration of the primary labeled LIBS mAb [33]. Because flow cytometers have the ability to discriminate between free and bound fluorescent molecules in a homogeneous assay [42], the concentration of the unlabeled ligand-receptor complex is proportional to the fluorescence of the fluorophores associated with the mAbs [54]. The major advantage of this approach, compared with direct binding of a fluorescent ligand, is that there is no limit to the ligand concentration. Because the LDV ligand is unlabeled, no non-specific fluorescence increase with ligand concentration is observed, and virtually no non-specific antibody binding is detected [33,41,54]. This assay allows the detection of integrin affinity changes that differ by several orders of magnitude [55].

Cells were incubated in the presence of increasing concentrations of unlabeled LDV and phycoerythrin-labeled anti-CD29 LIBS antibody (HUTS-21) in the presence or absence of the CO donor. As shown previously, the EC50 for mAb binding is similar to the ligand dissociation constant (K_d_) determined using other methods [54]. We observed that the EC50s for LIBS antibody binding for CORM-2 treated samples were larger than for the vehicle treated controls by ~2 fold for resting cells, and ~3 fold for FPR activated cells (Figure 6). This indicated a decrease in the ligand binding affinity. Also, the fact that the maximal binding of LIBS antibody for CORM-2 treated samples was ~5-10% lower was suggested by the change in VLA-4 receptor surface expression (CD49d/CD29 complex) as detected using anti-CD49d and anti-CD29 antibodies (Figure 5).

**Figure 7:**
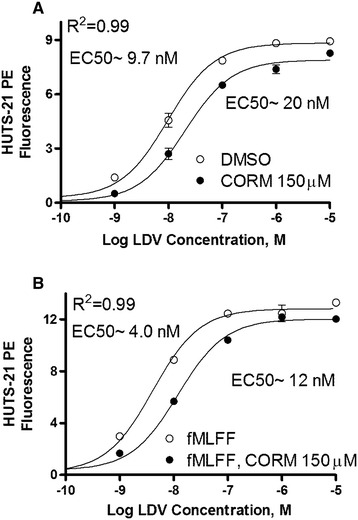
**Effect of CO donor on binding of primary labeled LIBS (HUTS-21) to U937 cells in the presence of different concentrations of LDV.** Mean fluorescence intensity plotted versus concentration of unlabeled LDV. **A**. U937 cells were treated with vehicle (DMSO, control), and CO donor (150 μM CORM-2) for 30 min at 37°C. Next, cells were incubated with the indicated concentration of LDV in the presence of excess HUTS-21 mAbs, washed, and fluorescence was measured. **B**. U937 cells stably transfected with the non-desensitizing FPR mutant were treated with vehicle (DMSO, control), and CO donor (150 μM CORM-2) for 30 min at 37°C in the presence of 100 nM fMLFF for cell activation. Next, mAb binding was performed in a manner analogous to A. The non-specific binding of HUTS-21, detected in the absence of LDV ligand, was subtracted from the data. Means ± SEM of three independent determinations are shown. The data were fitted using the sigmoidal dose–response equation with GraphPad Prism software. One representative experiment out of three independent experiments is shown.

To evaluate the effects of the CO donor on the affinity of the VLA-4 specific ligand we compared data obtained using fluorescent LDV-FITC (Figures 2A,C and 4A,C) with LDV binding detected using LIBS (Figure 7). Notice that the two highest concentrations of the CO donor or hemin induced very similar effects (see Figures 2A and 4A). The decrease in LDV-FITC binding on resting cells saturated at ~30% for both compounds. Quantitatively, in order to observe a decrease from 68% occupancy for the binding of the LDV-FITC ligand on resting cells (the initial binding occupancy as calculated based on resting VLA-4 K_d_ ~12 nM [30], and LDV-FITC concentration 25 nM) to ~30%, a ~4.8 fold increase in the K_d_ would be required (at K_d_ ~58 nM, ligand concentration 25 nM, receptor occupancy ~30% as derived from the one-site hyperbolic binding equation).

For FPR activated cells, a decrease from ~80% initial occupancy (K_d_ ~1 nM, and LDV-FITC concentration 4 nM), down to ~25% occupancy, requires a ~12 fold increase in the K_d_ (at K_d_ ~12 nM, ligand concentration 4 nM). This suggests that the effect of CO is more significant in the case of integrins activated through inside-out signaling. It is worth noting that K_d_ ~12 nM corresponds to the K_d_ detected on resting cells, and the real-time decrease in LDV-FITC binding after CORM-2 addition reached a plateau at a level that was close to the level corresponding to the resting cell (before FPR activation, Figure 2C). This observation additionally supports our calculations.

The effect of the CO donor on the ligand affinity change detected using the LIBS antibody was smaller. We detected only two to three fold differences in the EC50s (Figure 7). The EC50 for fMLFF activated cells (Figure 7B) was higher than the K_d_ estimated using dissociation rate analysis [30,35,56]. This discrepancy may be attributed to desensitization of FPR signaling after long-term incubation in the presence antibodies. This data additionally emphasizes the importance of rapid real-time approaches for studying cell signaling [33].

### The activator of soluble guanylyl cyclase and the cell permeable cGMP analog decrease the binding affinity of the VLA-4 specific ligand

The effects of BAY 41–2272, an activator of soluble guanylyl cyclase and dibutyrylguanosine 3’,5’-cyclic monophosphate, a cell permeable cGMP analog, (Figure 1) were studied previously as part of our study of nitric oxide signaling and VLA-4 regulation [8]. We found that BAY 41–2272 induced a rapid dose-dependent down-regulation of LDV-FITC binding after activation through CXCR4, CXCR2, and the non-desensitizing mutant of FPR. Cell treatment with BAY 41–2272 exhibited faster dissociation rates (k_off_) indicating a decrease of the affinity of the VLA-4 specific ligand. DbcGMP also induced rapid and dose dependent down-regulation of LDV-FITC binding [8]. Given the similarity between NO and CO signaling and their functional roles, both gases stimulate guanylyl cyclase [7,57]. These data support the role of the CO signaling pathway in regulating VLA-4 conformation and cell adhesion.

## Discussion

### Do cyclic nucleotides act as universal anti-adhesive integrin regulators?

During the last few years we have described two signaling pathways that can rapidly down-regulate the binding of the VLA-4 specific ligand and cell adhesion: the Gαs-coupled receptor signaling pathway and the NO/cGMP signaling pathway [8,34]. CO-mediated VLA-4 inactivation is described in this report. One common feature of these pathways is the modulation of nucleotide cyclases leading to up-regulation of cyclic nucleotide (cAMP and cGMP) levels. Similar downstream signaling mechanisms, related to nucleotide-dependent kinases [58] and other effectors, suggest the need for further evaluation of the role of cyclic nucleotide-dependent pathways in integrin-dependent cell de-adhesion. The literature reveals that these pathways can participate in cell mobilization, demargination, detachment, or deployment, all of which can be linked to a decrease of α4-integrin-dependent cell adhesion.

For Gαs-coupled GPCRs, such as the β2-adrenergic receptor or the H2-histamine receptor, specific ligand agonists and antagonists, as well as an adenylyl cyclase activator and a cell permeable cAMP analog were able to rapidly modulate the affinity of the integrin and cell adhesion after SDF-1/CXCR4 or FPR meadiated activation [34]. This signaling pathway may participate in demarginating nonclassical monocytes [59] and CD8+ effector T-cells [60], triggering rapid detachment of PBMCs attached to the endothelium [61], mobilizing natural killer cells [62-64], and playing a role in β2-adrenergic receptor and cAMP-induced leukocytosis [65-67]. However, further studies are needed to dissect the interplay between cytokines and this signaling pathway [68]. Rapid mobilization of hematopoietic stem progenitor cells (HSPCs) mediated specifically through VLA-4 adhesion and SDF-1/CXCR4-dependent signaling [55,69-75] was induced by β2-adrenergic receptor agonists, and can be blocked by a specific antagonist in mice [76]. We envision that β2-adrenergic receptor/cAMP-dependent VLA-4 deactivation [34] provides one of the molecular mechanisms for regulating stem progenitor cell egress from the bone marrow [77,78]. Another mechanism can be related to NO signaling.

Our discovery that the NO/cGMP signaling pathway rapidly deactivates high affinity VLA-4 pre-activated by SDF-1/CXCR4 signaling and other Gαi-coupled GPCRs [8] provides a plausible explanation for the observation that links NO-mediated signaling and nitric oxide synthase with stem and progenitor cell trafficking and mobilization. Mice deficient in endothelial nitric oxide synthase (NOS3 or eNOS) exhibited impaired mobilization of endothelial progenitor cells (EPC) from the bone marrow. The expression of CD29 (VLA-4 β1-subunit) and CXCR4 remained unaltered in NOS3^−/−^ mice, but neither the conformation nor affinity state of the integrin was tested [79]. Bone marrow stromal and vascular cells express large quantities of NOS. These cells are envisioned to be a major source of NO. Both stromal and vascular cells express VEGF-receptor-2 (Flk-I), and VEGF is one of the major factors mobilizing endothelial progenitors from bone marrow [80,81]. VEGF has been shown to activate NO release through AKT-dependent phosphorylation of eNOS [80,82-85]. Thus, NO-triggered deactivation of VLA-4 could complement VEGF-induced stem cell mobilization (see Figure 3 in [82]). It is possible that the effect of NO is specific to CD34^+^ Flk-I^+^ EPCs. Under NO-deficient conditions EPCs failed to be mobilized into the peripheral blood. The c-kit^+^Lin^−^ HSPCs were not affected by nitric oxide [86]. Thus, existing data support specific role of cyclic nucleotide-related signaling as a regulator of cell mobilization, demargination, or detachment under Gαs-coupled GPCRs or NO signalling.

### CO in macrophages

The effect of CO is typically attributed to the enzymatic catabolism of heme, and the majority of CO (up to ~70%) is produced from hemoglobin originating from the breakdown of erythrocytes [20]. Splenic red-pulp macrophages are responsible for the removal of senescent red blood cells from the circulation, during filtration of blood though the spleen [87]. These macrophages express heme oxygenase-1 (HO-1) that metabolizes heme and produces iron, biliverdin, and carbon monoxide [4]. HO-1 is essential for splenic macrophage function, since in HO-1^−/−^ mice macrophages are destroyed through exposure to unmetabolized heme [88]. At the same time, the spleen is reported to serve as a reservoir for resident macrophages that can be rapidly deployed in the peripheral blood in response to surgically induced ischemia of the myocardium [89]. The data presented in this report suggest a possible mechanism for the deployment phenomenon.

Erythrocyte damage can be induced by a number of factors that include ischemia and inflammation. This may lead to the increased clearance of red blood cells [90]. As a result, increased phagocytosis by splenic macrophages can lead to rapid up-regulation of heme catabolism and intra-macrophage CO production. This is expected to down-modulate integrin affinity and cell adhesion within the phagocyte. It is also possible that CO can diffuse from the phagocyte and act in a paracrine manner on macrophages residing in close proximity. The loss of cell adhesion could result in macrophages entering the circulation through the efferent splenic vein. This scenario seems plausible specifically because of the role of α4-integrins in splenic homing [87,91].

Another possible implication of the anti-adhesive impact of CO signaling is in ischemia-reperfusion injury and transplantation [4]. Adhesion molecules and specifically β1- and β2- integrins are envisioned as feasible targets for preventing reperfusion injury through blocking leukocyte extravasation and recruitment [92-94]. The successful application of CO for organ transplant could be accompanied by reduced recruitment of leukocytes, macrophages, and T-cells to the graft [95], and the overall anti-inflammatory effect of CO could also be related to blocking integrin-dependent immune cell adhesion [4]. Thus, it seems that the enzymatic catabolism of heme in marcrophages and resulting intra-macrophage CO production are critical for rapid down-regulation of integrin-mediated cell adhesion, rapid re-entry of adherent cells into circulation, or blocking of leukocyte extravasation and recruitment to the sites of inflammation. We propose that therapeutic modulation of this pathway can serve as a viable alternative to a direct modulation of cell adhesion using integrin antagonists for example.

### CO, integrins, and immune response

The release and accumulation of hemin in peripheral blood can result from hemolysis that occurs as a consequence of bacterial infection [96]. Free heme plays a crucial role in the pathogenesis of sepsis [97]. Since VLA-4 and VLA-5 are known to regulate phagosome maturation and microorganism clearance in macrophages [98], the pathogen-induced damage of erythrocytes, which decreases integrin binding through activation of the CO/cGMP pathway, should promote pathogen survival. In particular, the lipopolysaccharide-binding pattern recognition molecule mindin (spondin-2) specifically interacts with CD49d/CD29 [99] though the LEV (Leu-Glu-Val) integrin-binding motif [100] homologous to the LDV (Leu-Asp-Val) binding motif in fibronectin [101]. Mindin is essential for microorganism clearance because mindin-deficient macrophages show defective phagocytosis [102]. Therefore, one would expect that the binding of mindin to VLA-4 will be regulated by CO in a manner similar to that of the LDV probe, described in the current report. Thus, a release of large amount of free heme and a subsequent CO production in macrophages will provide a protection of pathogens against microorganism clearance. However, no significant effect of exogenous heme administration on the number of bacterial colony-forming units in the blood and peritoneum of mice subjected to a nonlethal polymicrobial infection were found, and the ability of heme to precipitate sepsis was not directly related to the pathogen load [97]. Nevertheless, because hemolysis can be caused by pore-forming toxins produced by many blood-borne pathogens [103], the amount of free heme should be directly related to the bacterial load. The pore forming toxins are known to contribute to the evasion of host defence by the inhibition of innate immune responses in macrophages [104]. Thus, a number of immunological phenomena related to pathogen-induced hemolysis can be related to the regulation of VLA-4 integrin-mediated binding of pattern recognition molecules, phagosome maturation and microorganism clearance in macrophages. The ability to release free heme, induce production of large amount of CO, and thus block integrin-dependent cell adhesion could be one of the previously unrecognised immune evasion mechanisms employed by haemolytic pathogens.

## Conclusions

We conclude that CO from an artificial donor or a natural CO source (hemin) down-modulated binding of the VLA-4 integrin-specific ligand at rest and after inside-out activation through several Gα_i_-coupled receptors activation. This results in a rapid down-modulation of integrin-dependent cell adhesion. We propose that CO-triggered integrin deactivation represents a novel mechanism that provides a molecular basis for several phenomena related to the mobilization of different cell subsets, and the evasion of the immune response.

## Methods

### Materials

The VLA-4-specific ligand 4-((N’-2-methylphenyl)ureido)-phenylacetyl-L-leucyl-L-aspartyl-L-valyl-L-prolyl-L-alanyl-L-alanyl-L-lysine (LDV) and its FITC-conjugated analog (LDV-FITC probe) [30,33] were synthesized at Commonwealth Biotechnologies. Mouse anti-human CD29/integrin β_1_ chain, clone MAR4 (PE), mouse anti-human CD49d/ integrin α_4_ chain, clone 9F10 (PE), and isotype control (mouse IgG1 κ, PE) clone MOPC-21 were purchased from BD Biosciences and used according to the instructions of the manufacturer. Human recombinant CXCL12/SDF-1α and recombinant human CXCL8/IL-8 were obtained from R&D Systems, Inc. All other reagents were from Sigma-Aldrich. Stock solutions were prepared in DMSO, at concentrations ~1000-fold higher than the final concentration. Usually, 1 μl of stock solution was added to 1 ml of cell suspension, yielding a final 0.1% DMSO concentration. Control samples were treated with an equal amount of pure DMSO (vehicle).

### Cell lines and transfectant construct

The human histiocytic lymphoma cell line U937 and mouse melanoma cell line B78H1 were purchased from ATCC. Wild type CXCR4 (CD184) receptor-stably transfected U937 cells, wild type CXCR2 (IL8RB)-stably transfected U937 cells, and the non-desensitizing FPR ΔST mutant in U937 cells were prepared as described [105], and were a gift of Dr. Eric Prossnitz (University of New Mexico). For transfection of B78H1 cells, full-length human VCAM-1 cDNA was a kind gift from Dr. Roy Lobb of Biogen. The original construct [106] was subcloned into the pTRACER vector (Life Technologies Corp.). Transfection into B78H1 was done using the Lipofectamine transfection reagent (Life Technologies Corp). High receptor-expressing cells were sorted using a MoFlo flow cytometer (Beckman Coulter Inc.). Cells were grown at 37°C in a humidified atmosphere with 5% CO2 and 95% air in RPMI 1640 (supplemented with 2 mM L-glutamine, 100 units/ml penicillin, 100 μg/ml streptomycin, 10 mM HEPES, pH 7.4, and 10% heat-inactivated fetal bovine serum). Cells were counted using the Coulter Multisizer/Z2 analyzer (Beckman Coulter Inc.). For experiments, cells were suspended in warm RPMI (37°C) at 1 × 10^6^ cells/ml and used immediately.

### Kinetic analysis of binding and dissociation of VLA-4 specific ligand

Kinetic analysis of the binding and dissociation of the LDV-FITC probe was described previously [30,33]. Briefly, cells (1 × 10^6^ cells/ml) were preincubated in warm RPMI under the appropriate conditions for 10–20 min at 37°C. Flow cytometric data were acquired for up to 1024 s at 37°C while the samples were stirred continuously at 300 rpm with a 5 × 2 mm magnetic stir bar (Bel-Art Products). Samples were analyzed for 30–120 s to establish a baseline. The fluorescent ligand was added and acquisition was re-established, creating a 5–10 s gap in the time course. For real-time affinity activation experiments, 4 nM LDV-FITC was added after establishing a baseline. Then, data were acquired for 2–3 minutes, and cells were treated with different GPCR ligands at saturating concentration (10 times or higher than K_d_). In several experiments, cells were treated sequentially with two or more different compounds. Acquisition was re-established, and data were acquired continuously for up to 1024 s.

The concentration of the LDV-FITC probe used in activation experiments (4 nM) was below the dissociation constant (K_d_) for its binding to resting VLA-4 (low affinity state, K_d_ ~ 12 nM), and above the K_d_ for physiologically activated VLA-4 (high affinity state, K_d_ ~ 1–2 nM) [30]. Therefore, the transition from the low affinity to the high affinity receptor state led to increased binding of the probe (from ~25% to ~70 – 80% of receptor occupancy, as calculated based on the one site binding equation), which was detected as an increase in the mean fluorescence intensity. For kinetic dissociation measurements, cell samples were preincubated with the fluorescent probe (25 nM), treated with excess unlabeled LDV (2 μM), and the dissociation of the fluorescent molecule was followed. The resulting data were converted to mean fluorescence intensity versus time using FCSQuery software developed by Dr. Bruce Edwards (University of New Mexico).

### Cell aggregation assay

The cell suspension aggregation assay has been described previously [35,37]. Briefly, U937 cells were labeled with the green fluorescence PKH67GL dye, and B78H1/VCAM-1 transfectants were stained with the red fluorescence PKH26GL dye (Sigma-Aldrich). Labeled cells were washed, resuspended in RPMI and stored on ice until used in the assays. Control U937 cells were preincubated with the LDV molecule for blocking. Prior to data acquisition, cells were warmed to 37°C for 10 min separately and then mixed. During data acquisition, the samples were stirred with a 5 × 2-mm magnetic stir bar (Bel-Art Products, Pequannock, NJ) at 300 rpm and kept at 37°C. The number of cell aggregates containing red and green co-fluorescent particles, and the number of singlets were followed in real-time. The percentage of aggregates (Agg, %) was calculated as follows: Agg, % = (number of aggregates/(number of aggregates + number of singlets)) × 100. Experiments were performed using a FACScan flow cytometer and Cell Quest software (Becton Dickinson, San Jose, CA). The real-time aggregation kinetic data were converted to Agg, % versus time using FCSQuery software developed by Dr. Bruce Edwards (University of New Mexico). Figures were prepared using Summit V4.3 software (Beckman Coulter Inc.).

### Statistical analysis

Curve fits and statistics were performed using GraphPad Prism version 4.00 for Windows, GraphPad Software, San Diego California USA, www.graphpad.com. Each experiment was repeated two or three times. The experimental curves represent the mean of two or more independent runs. SEM was calculated using GraphPad Prism.

## Abbreviations

CO: Carbon monoxide
CORM-2: Carbon monoxide-releasing molecule 2, tricarbonyldichlororuthenium (II) dimer
dbcAMP: N-6,2’-O-dibutyryladenosine 3’,5’-cyclic monophosphate
EPC: Endothelial progenitor cell
FITC: Fluorescein isothiocyanate
GC: Guanylyl cyclase
fMLFF: N-formyl-L-methionyl-L-leucyl-L-phenylalanyl-L-phenylalanine, formyl peptide
FPR: Formyl peptide receptor 1
GPCR: Guanine nucleotide binding protein coupled receptor
HEPES: 4-(2- hydroxyethyl)-1-piperazineethanesulfonic acid
HO: Heme oxygenase
HSPC: Hematopoietic stem progenitor cell
LDV: 4-((N’-2-methylphenyl)ureido)-phenylacetyl-L-leucyl-L-aspartyl-L-valyl-L-prolyl-L-alanyl-L-alanyl-L-lysine
LDV-FITC: 4-((N’-2-methylphenyl)ureido)-phenylacetyl- L-leucyl-L-aspartyl-L-valyl-L-prolyl-L-alanyl-L-alanyl-L-lysine-FITC
LIBS: Ligand induced binding site
mAb: Monoclonal antibody
MCF: Mean channel fluorescence, equivalent of mean fluorescence intensity
NOS3 or eNOS: Endothelial nitric oxide synthase
SDF-1: Stromal cell-derived factor-1, CXCL12
VCAM-1: Vascular cell adhesion molecule 1, CD106
VLA-4: Very late antigen 4, CD49d/CD29, α4β1 integrin
VLA-5: Very late antigen 5, CD49e/CD29, α5β1 integrin
